# Supplementary material for: Southern Madagascar, polycrisis and project failures: A scoping review
Source: PLoS One. 2024 Jul 25;19(7):e0305359. doi: 10.1371/journal.pone.0305359 (PMC11271851; doi:10.1371/journal.pone.0305359)
Supplement: S1 File — (PDF) [file pone.0305359.s002.pdf]

Supplementary file PubMed search equation

"Madagascar"[Title/Abstract] AND ("south"[Title/Abstract] OR "sud"[Title/Abstract] OR "androy"[Title/Abstract] OR "atsimo"[Title/Abstract] OR "anosy"[Title/Abstract]) AND ("climat"[All Fields] OR "environment"[All Fields] OR "environnement"[All Fields] OR "ressource"[All Fields] OR "defor"[All Fields] OR "water"[All Fields] OR "eau"[All Fields] OR "aqua"[All Fields] OR "agri"[All Fields] OR "fish"[All Fields] OR "peche"[All Fields] OR "rear"[All Fields] OR "elevage"[All Fields] OR "alimentation"[All Fields] OR "nutrition"[All Fields] OR "food"[All Fields] OR "undernourishment"[All Fields] OR "genre"[All Fields] OR "gender"[All Fields] OR "femme"[All Fields] OR "women"[All Fields]) AND ("development"[All Fields] OR "developpement"[All Fields] OR "fail"[All Fields] OR "echec"[All Fields] OR "project"[All Fields] OR "projet"[All Fields]) AND ("institution"[All Fields] OR "cultu"[All Fields] OR "socio"[All Fields] OR "anthropo"[All Fields] OR "system"[All Fields] OR "politic"[All Fields] OR "state"[All Fields] OR "etat"[All Fields] OR "gouv"[All Fields]) 1990:2023 [dp]

Note: the search was carried out on 14/06/2023.
